# Frequency of Yoga Practice Predicts Health: Results of a National Survey of Yoga Practitioners

**DOI:** 10.1155/2012/983258

**Published:** 2012-08-14

**Authors:** Alyson Ross, Erika Friedmann, Margaret Bevans, Sue Thomas

**Affiliations:** ^1^University of Maryland School of Nursing, 655 West Lombard Street, Baltimore, MD 21201, USA; ^2^National Institutes of Health Clinical Center, 10 Center Drive, Room 2B13, MSC 1151, Bethesda, MD 20892, USA

## Abstract

*Background*. Yoga shows promise as a therapeutic intervention, but relationships between yoga practice and health are underexplored. *Purpose*. To examine the relationship between yoga practice and health (subjective well-being, diet, BMI, smoking, alcohol/caffeine consumption, sleep, fatigue, social support, mindfulness, and physical activity). *Methods*. Cross-sectional, anonymous internet surveys distributed to 4307 randomly selected from 18,160 individuals at 15 US Iyengar yoga studios; 1045 (24.3%) surveys completed. *Results*. Mean age 51.7 (± 11.7) years; 84.2% female. Frequency of home practice favorably predicted (*P* < .001): mindfulness, subjective well-being, BMI, fruit and vegetable consumption, vegetarian status, sleep, and fatigue. Each component of yoga practice (different categories of physical poses, breath work, meditation, philosophy study) predicted at least 1 health outcome (*P* < .05). *Conclusions*. Home practice of yoga predicted health better than years of practice or class frequency. Different physical poses and yoga techniques may have unique health benefits.

## 1. Introduction

Three out of every four dollars in health care are spent on treatment of chronic, lifestyle-related health conditions including obesity, type 2 diabetes, cardiovascular disease (CVD), and cancer [1]. These conditions are associated with a number of modifiable health behaviors including diet [2, 3], physical activity [4–6], cigarette smoking [7, 8], and excessive alcohol consumption [9, 10]. Poor mental health [11], low social support [12, 13], and poor sleep [14] are also factors that contribute to morbidity and mortality. 

Making even small, positive changes in health behaviors significantly improves mortality rates [15], and simultaneously changing multiple health behaviors results in further reductions in morbidity and mortality [16, 17]. While the answer to America's health crisis appears clear, permanently improving health behaviors has proven to be elusive. 

Yoga, an ancient discipline that uses a combination of practices including physical poses, breath work, and meditation, is defined by Patanjali in the second yoga sutra as “the stilling of the changing states of the mind [18].” It has recently shown promise as an intervention targeting a number of outcomes associated with lifestyle-related health conditions including cardiovascular disease [19], metabolic syndrome [20], diabetes [21], and cancer [22]. While aerobic exercise long has been a valuable tool in combating these health conditions, a review of clinical trials comparing exercise to yoga found yoga to be equal or superior to aerobic exercise in improving a number of outcomes associated with chronic health conditions [23].

One mechanism that would explain the effectiveness of yoga interventions compared with exercise interventions is that, in addition to the benefits of increased physical activity associated with the physical practice of yoga poses, yoga appears to downregulate the Hypothalamic-Pituitary-Adrenal (HPA) axis and the Sympathetic Nervous System (SNS) response to stress, possibly via direct vagal stimulation [20]. Repeated firing of the HPA axis and SNS can lead to dysregulation of the system and ultimately diseases such as obesity, diabetes, autoimmune disorders, depression, substance abuse, and cardiovascular disease [24, 25]. Numerous studies have shown yoga to have an immediate download effect on both the SNS/HPA axis response to stress by decreasing cortisol [26, 27] and blood glucose [28, 29], as well as norepinephrine and epinephrine levels [30]. Yoga significantly decreases heart rate, systolic and diastolic blood pressure [30–32], and inflammation [33], and yoga increases levels of Immunoglobulin A [34] and Natural Killer Cells [35]. In addition to the immediate SNS and HPA-axis effects, yoga improves outcomes associated with chronic SNS/HPA-axis activation: blood cholesterol [36–38]; body composition including: BMI [39], body weight [36, 37, 40], and waist circumference [29]; fatigue [41, 42]; and sleep in healthy and diseased populations [43].

While this evidence is promising, the relationship between yoga practice and positive changes in health behaviors remains unclear. Only three studies were found examining relationships between yoga practice and aspects of health in individuals who practice yoga [40, 44, 45]. These studies contributed valuable evidence that there may be a favorable relationship between regular yoga practice and BMI [44, 45], diet [45], and weight maintenance [40]. However, these studies looked only at yoga practice in general and did not examine the relative contributions of the different aspects of yoga practice. 

This also appears to be the case in many clinical trials involving yoga, as studies frequently focus either exclusively on interventions using only physical poses or breath work, or they utilize a combination of different aspects of yoga practice (including vigorous physical poses, gentle restorative poses, breath work, and meditation) without examining the relative contributions of the individual aspects. This creates two problems when interpreting the results. First, when yoga is taught according to classical texts such as the Yoga Sutras [18], it consists of eight components; only one of the eight components focuses on the physical practice of poses. The remaining components consist of breath work, control of the mind and senses, meditation, and ethical practices that guide one's behaviors such as how to interact with others and how to treat oneself [18]. A second reason for examining the relative contributions of the different aspects of yoga practice is that the different physical poses (standing poses, vigorous poses such as arm balances and back bends, inversions such as head stand and should stand) and yoga practices such as breath work and meditation are believed to have different physiological and psychological effects [46]. No studies were found in peer-reviewed journals that examine the relative contributions of the various aspects of yoga. Therefore, the clinical value of the individual components remains unclear. 

The objective of this study is to better understand the interrelationship between yoga practice and health. Specifically, the study addressed the contributions of yoga practice in general (years of practice, classes per month, and/or days per month of home practice) and practice of specific components of yoga practice (physical poses, breath work, meditation, and/or philosophy study) to these aspects of health. It is important to study the unique contributions of the individual components of yoga practice because some aspects of yoga practice may be more effective than others in improving specific health outcomes such as body weight, sleep, and mental health. 

## 2. Methods

The study utilized a cross-sectional design with an anonymous online survey to examine yoga practice and its relationship to aspects of health including physical activity, fruit and vegetable consumption, sleep disturbance, fatigue, social support, mindfulness, and subjective well-being. 

### 2.1. Participants and Randomization

Approval for the study was obtained from the Institutional Review Board at the University of Maryland, Baltimore. Individuals included (1) were at least 18 years of age, (2) practiced yoga (either taking classes or practicing at home) at least weekly for a minimum of two months within the past 6 months, and (3) had Internet access and ability to complete an online survey. The length and amount of yoga practice required to be included in the study was chosen based upon the expert opinion of Senior Iyengar yoga instructors as to the minimum required to be considered an individual who practices yoga. 

The researchers worked with the Iyengar Yoga National Association U.S. (IYNAUS) in selecting study participants. Iyengar yoga studios were chosen because they have (1) a large national organization (representing over 900 teachers and 100+ studios) and (2) strict standardization of teaching that would likely contribute to consistent instruction. 

 There are over 900 certified Iyengar Yoga Teachers within IYNAUS, representing over 100 yoga studios. The investigators worked with IYNAUS to target studios in the major geographic regions (Northeast, Southeast, Midwest, Southwest, and West), taking steps to ensure all the regions were represented in proportion to the number and size of studios in their region. Based on an a priori power analysis, 15 studios with e-mail list serves of 18,160 were selected to participate in the survey. Random sampling software (SPSS version 19) was used to draw a random sample (approximately 25%) of e-mail addresses from each studio. Using this sampling strategy, 4307 potential subjects were randomly selected from the 15 yoga studios to receive a secure link to the survey, which was sent by the studio owners. Of the 1164 individuals (27%) who responded to the survey, 1045 (89.8%) completed the survey in its entirety and met inclusion criteria. Data were collected from June to September of 2011. 

### 2.2. Measurement

Following strategies suggested by Dillman et al. [47] to develop and implement the survey, the researchers used SurveyMonkey to create a 65-item questionnaire that asked detailed questions about yoga practice and health. Health outcomes were aspects of health that are associated with increased risk of morbidity and mortality. The survey utilized preexisting measures except for demographic characteristics, descriptors of yoga practice, and a few individual health items including smoking, alcohol consumption, and vegetarian status. 

#### 2.2.1. Yoga Practice

Questions regarding yoga practice were divided into questions about general yoga practice (years of practice and frequency of home practice and yoga classes) and specific components of yoga practice (physical poses, breath work, meditation, and philosophy study). Physical poses were divided into four categories: standing poses; vigorous poses such as sun salutations, backbends, and arm balances; inversions such as head and shoulder stands; gentle and/or restorative poses. Frequency of physical poses was defined as days per month of practice at home and in class, with the exception of gentle poses, which was defined as ≤30 or >30 minutes per week. Frequency of breath work and meditation were defined as ≤ or > once per week. The study of yogic philosophical texts, primarily the Yoga Sutras of Patanjali, is considered one of the ethical requirements of yoga study [18]. The amount of time spent on the study of yoga philosophy (yoga sutras) was defined as the frequency with which one attends classes or lectures (including recordings or webcasts) or reads classical yoga texts such as the Yoga Sutras, Bagavad Gita, or the Upanishads. 

#### 2.2.2. Demographics

Demographic data were collected from each subject including information regarding: age, gender, race, height, weight, education, marital status, and job status. Body mass index (BMI) was calculated using the following formula: [Weight (pounds)/height (inches) 2] × 703 [48].

#### 2.2.3. Sleep Disturbance, Fatigue, and Social Support

Sleep disturbance (4-items), fatigue (4-items), and social support (8-items) were measured using short forms from the Patient-Reported Outcomes Measurement Information System (PROMIS) (sleep disturbance and fatigue) and the National Institutes of Health (NIH) Toolbox (social support). PROMIS and the NIH Toolbox are initiatives of the NIH designed to provide the public with a free national item bank of valid and reliable measures of commonly used patient-reported outcomes measures [49, 50]. All item banks have high reliability [51] and compare favorably with legacy measures [52]. Each item is assessed on a 5-point Likert scale, ranging from 1 (“not at all”) to 5 (“very much”) to measure perceptions of the amounts of social support (during past month), as well as sleep disturbance and fatigue (during past 7 days), with higher scores indicating of higher levels of the concepts. In the present study, Cronbach's alpha was .83 for sleep disturbance, .90 for fatigue, and .96 for social support.

#### 2.2.4. Subjective Well-Being (Happiness)

Subjective well-being is a multidimensional construct of mental health involving emotional, psychological, and social well-being, often referred to as “happiness [53].” Subjective well-being was measured using the 14-item Mental Health Continuum-short form (MHC-SF) that asked subjects to report how frequently they experienced symptoms of positive mental health in the past month. Answers range from “never” to “every day” and scores range from 14 to 70, with higher scores indicating higher levels of subjective well-being. Cronbach's alpha for the total scale was .91 in the present study. 

#### 2.2.5. Fruit and Vegetable Consumption

The number of servings per day of fruits and vegetables was obtained using 7 items from the National Cancer Institute's Multifactor Screener, a self-report, food frequency questionnaire. The Multifactor Screener asked subjects how often they ate fruits and vegetables during the past month. Responses ranged from never to several times per day, from which pyramid servings of fruits and vegetables per day were calculated. This questionnaire was validated in a number of large studies including NCI's Observing Protein and Energy (OPEN) study and Eating at America's Table Study (EATS), with correlations with true consumption ranging from 0.5 to 0.8 [54].

#### 2.2.6. Physical Activity

Information regarding physical activity was gathered using the 7-item International Physical Activity Questionnaire (Short form) (IPAQ). Subjects were instructed to report average number of days per week and minutes per day of physical activity, not including yoga classes or practice, during the past month. Results were used to calculate the total number of metabolic-equivalent minutes (MET-min) of exercise per week and levels of physical activity [55]. The IPAQ was extensively studied in 12 countries and was found to be valid and reliable in 18 to 65-year-old adults in a variety of settings [56, 57].

#### 2.2.7. Freiberg Mindfulness Inventory—Short Form

Mindfulness was measured using the Freiberg Mindfulness Inventory—Short Form (FMI-SF), an 8-item version of the original 30-item Freiberg Mindfulness Inventory [58, 59] that uses a 4-point Likert scale to assess how frequently subjects experience certain situations or mind states. Scores range from 8 to 32, with higher scores indicating higher levels of mindfulness. Cronbach's alpha was .87 in the present study.

#### 2.2.8. Other Health Information

Single items were used to assess current smoking status (yes/no), vegetarian status (defined as no consumption of meat, fish, or poultry), and alcohol consumption (alcoholic drinks per week).

### 2.3. Statistical Analysis

Data cleaning techniques using SPSS 19.0 were used to identify miscoded data, outliers, and missing data. Because the survey required participants to answer every question, less than 5% of the data were missing. Incomplete cases were excluded from future analyses. Independent *t* tests were used to examine differences between those cases with and without missing data as well as incomplete versus complete cases; no significant differences were noted. Three variables (gentle poses, meditation, and breath work) were badly skewed and could not be normalized. These three variables were dichotomized using the median as a cut point. Descriptive statistics (frequencies, percentages, measures of central tendency, and standard deviations) were obtained to describe the demographic data, yoga practice habits, and aspects of health. According to Gellman and Hill [60], analysis adjustment for multiple analyses is not necessary in an exploratory model building context. Thus, the researchers used a .05 level of significance for all analyses. 

Research questions examining relationships of predictors with outcomes were analyzed using linear or logistic regression, depending upon the level of measurement of the outcome. Regression analyses were conducted using the following steps. First, bivariate relationships of yoga practice and demographic variables with aspects of health were investigated by computing Pearson *r* correlations. Next, any yoga practice variable or demographic variable that had at least a small (*r* = .10) and significant relationship to the health variable of interest was included in appropriate regression analyses (linear or logistic) to examine the independent effects of all correlated variables [61]. Variables that were significant at *P* = .05 were placed into subsequent regressions to examine interaction effects. Interactions that were not significant at *P* = .05 were removed, one at a time, until the final model was determined. 

Research questions examining differences in means between those with high and low practice frequency were analyzed using independent *t* tests. Cut off points for determining high and low yoga practice groups were selected using the highest and lowest quartiles. A priori power analyses showed the 1045 cases were sufficient to achieve 80% power with alpha .05 with a medium effect size for all analyses. 

## 3. Results

Demographic characteristics of the study sample are included in Table 1. The age of participants ranged from 19 to 87 years (*M* = 51.7 ± 11.7). The large majority of subjects was female (84.2%) and white (89.2%). Most of the subjects were married (61.3%) and employed full time (50.9%). They were highly educated, with almost 90% having either an undergraduate (36.9%), master's (37%), or a doctoral (13.5%) degree. Subjects reported practicing yoga for less than one to more than 25 years (*M* = 11.4 ± 7.5). They reported taking between zero and 28 classes per month (*M* = 6.1 ± 5.1) and practicing yoga outside of class up to 28 days per month (*M* = 12.2 ± 9.7).

**Figure 1:**
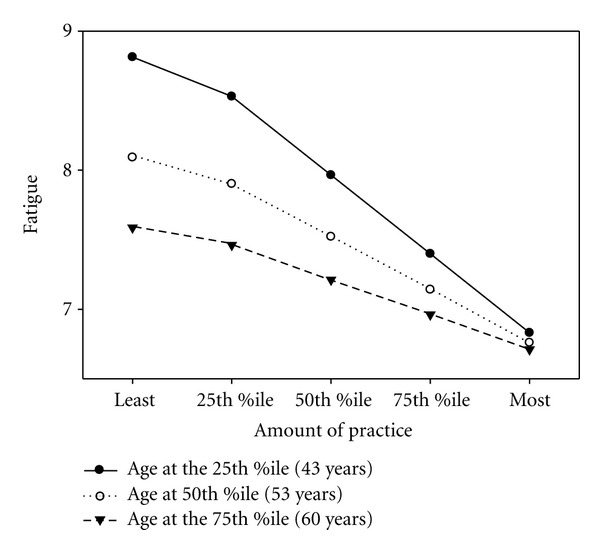
Interaction effect between frequency of home yoga practice and age (*n* = 1043). Note: Percentiles for frequency of home yoga practice: least = 0 days/month, 25th = 4 days/month, 50th = 12 days/month, 75th = 20 days/month, most = 28 days/month.

In the final models examining general yoga practice, frequency of home practice was the practice variable that most often predicted aspects of health (Table 2). Specifically practice frequency (*β* = .106, *P* < .001) and years of practice (*β* = .039, *P* < .05) were independent predictors of mindfulness. For every extra day per week of yoga home practice, mindfulness scores increased .42 of a point (.10 of a SD). After controlling for gender and age, practice frequency was a significant independent predictor of subjective well-being (*β* = .183, *P* < .001) and BMI (*β* = −.043, *P* < .001). Every additional day per week of home practice was associated with a decrease of .17 of a point (.04 of SD) in BMI. After controlling for gender and age, practice frequency predicted fruit and vegetable servings per day (*β* = .031, *P* < .001). Practice frequency was the only variable negatively related to sleep disturbance (*β* = −.052, *P* < .001), and individuals who practiced more frequently had higher odds of being a vegetarian than those who practiced less often (OR = 1.057, *P* < .001). For every additional day per week of yoga practice, sleep improved by .21 of a point (.07 of an SD) and the odds of being vegetarian increased 22.8%. After controlling for the effects of practice frequency (*β* = −.171, *P* < .01) and age (*β* = −.072, *P* < .01), there was a significant interaction effect between practice frequency and age on fatigue (*β* = .002, *P* < .01). Older individuals had lower levels of fatigue regardless of practice frequency, but younger individuals with a higher frequency of home practice exhibited lower levels of fatigue than those who practiced less often (see Figure 1). At the highest levels of practice, older and younger individuals experienced similar fatigue levels.

Because practice frequency was such an important predictor of health, the authors explored differences in yoga practice between intense practitioners (those who practice at home ≥5 days per week) and less intense practitioners (those with a home practice of ≤1 day/week). Intense practitioners reported significantly more years of yoga practice (*M* = 15.1 ± 6.7 years versus 8.6 ± 7.2 years) than those who were less experienced (*t* = −12.038, *df* = 480; *P* < .001). Intense practitioners practiced more standing poses (*M* = 17.8 ± 7.6 days per month versus *M* = 7.2 ± 5.4 days per month; *t* = −21.587, *df* = 700; *P* < .001), more vigorous poses (*M* = 14.6 ± 8.5 days per month versus *M* = 4.3 ± 4.6 days per month; *t* = −19.755, *df* = 482.31; *P* < .001), and more inversions (*M* = 18.04 ± 9.0 days per month versus *M* = 4.51 ± 4.6 days per month; *t* = −24.508, *df* = 468.71; *P* < .001) than less intense practitioners. Those with high practice frequency report studying philosophy about once per month, compared to those with low practice frequency who study yoga philosophy only about 3 or 4 times per year (*P* < .001). Intense practitioners had nine times the odds of regularly practicing gentle poses, twice the odds of meditating at least weekly, and nearly three times the odds of practicing breath work at least weekly than those who reported low practice frequency (*P* < .001). 

In the final models examining specific components of yoga practice, all of the specific components predicted at least one aspect of health (Table 3). Notably, frequency of philosophy study was the yoga practice variable that most often predicted health. Frequency of philosophy study (*β* = .310, *P* < .001), along with breath work (*β* = .290, *P* < .001) and meditation (*β* = .282, *P* < .001), positively predicted mindfulness. Frequency of philosophy study (*β* = .756, *P* < .001), in addition to meditation (*β* = 2.80, *P* < .001) and female gender (*β* = 3.39, *P* < .001), also positively predicted subjective well-being. More frequent philosophy study also contributed to a lower BMI (*β* = −.158, *P* < .05) and higher odds of being a vegetarian (*β* = .242, *P* < .001). 

After controlling for gender, vigorous poses remained an independent predictor of BMI (*β* = −.053, *P* < .05). Vigorous poses also predicted sleep disturbance (*β* = −.065, *P* < .05). For every additional day per week of vigorous pose practice, BMI decreased .21 of a point (.05 of a SD) and sleep disturbance improved .26 of a point (.087 of an SD). Frequency of gentle poses (*β* = .360, *P* < .001), along with standing poses (*β* = .024, *P* < .01), remained positive predictors of fruit and vegetables consumption, even when controlling for the effects of age and gender. Those individuals who practiced gentle poses 30 minutes or more per week had 7% higher odds of being vegetarian (OR = 2.073, *P* < .01) and about 50% lower odds of consuming alcohol (OR = .621, *P* < .001) than those who practiced gentle poses for 30 minutes or less per week.

## 4. Discussion

In general, frequency of yoga practice outside of class, as opposed to years of yoga practice or class participation, was repeatedly a predictor of aspects of health including mindfulness, subjective well-being, BMI, fruit and vegetable consumption, and sleep disturbance. It did not appear to matter how long an individual had practiced yoga. Rather, it appeared to matter how often they practiced. While class participation may be important in learning to do yoga, it did not predict any aspects of health. Perhaps time spent in class counts as additional practice time, and it is not unique unto itself. 

 While the individual effects of frequency of home practice are small, accounting for less than 7% of the variance in the health variables, they are cumulative. For instance, for an individual who did not previously have a home practice of yoga, practicing one day per week is associated with consuming an extra tenth of a serving of fruits and vegetables per day or almost one extra serving per week. If that same individual were to practice five days per week, that would be associated with an increase of over one half a serving of fruits and vegetables per day or nearly four and a half extra servings per week. 

Individuals who were intense practitioners (5+ days per week of home practice) tended to practice all aspects of yoga including all of the physical poses, breath work, meditation, and philosophy study more often than those who did not practice as often. Intense practitioners attended class at the same rate as less intense practitioners. This possibly explains why class frequency did not predict any aspects of health. Because frequency of home practice was an important predictor of many aspects of health, and intense practitioners tended to practice all aspects of yoga, it is logical to assume that a practice that includes all aspects of yoga may be more beneficial to health than practice that includes only one or two aspects of yoga (such as only breath work or vigorous poses). 

The interaction effect of practice frequency with age on fatigue showed that older individuals, regardless of their practice frequency, had significantly lower levels of fatigue than younger yoga practitioners; younger individuals who had a more frequent home practice had less fatigue than younger practitioners who did not practice as often. Perhaps older individuals experience benefits from just a little bit of yoga practice, while younger individuals need more to experience benefits on fatigue. This finding is encouraging for older individuals beginning the practice of yoga, as problems with sleep and fatigue are common in the elderly [62, 63].

While no single category of physical pose (standing poses, vigorous poses, inversions, and/or gentle poses) was related to all aspects of health, each category of physical pose was related significantly to at least one aspect of health. The physical poses, often referred to as the “external” or physical practice in yoga texts [64], were most commonly related to the physical aspects of health (sleep, diet, BMI). In contrast, the higher level practices of breath work and meditation, typically defined in yoga texts as tools for controlling a distracted, fluctuating mind [64], were associated with mindfulness and subjective well-being. It is possible that physical poses, particularly active poses (such as standing poses) and vigorous poses (such as sun salutations, backbends, and arm balances), have effects similar to those of exercise. These findings support previous evidence that exercise is related to diet [65], energy levels [65], and BMI [66]. Levels of physical activity in this population predicted fruit and vegetable consumption and levels of fatigue, although the effects (standardized betas) of physical activity were smaller than those of yoga home practice. Likewise, because breath work and meditation appear to influence mindfulness and well-being, they may be particularly useful in treating conditions such as depression and anxiety.

More frequent practice of gentle poses, including supine restorative poses and relaxation pose (Savasana), were associated with three aspects of health that deal with feeding behaviors or cravings: higher fruit and vegetable consumption, higher rates of vegetarianism, and lower alcohol consumption. It has been postulated that yoga impacts the Hypothalamic-Pituitary-Adrenal (HPA) axis and the Sympathetic Nervous System (SNS) response to stress [23], possibly via direct vagal stimulation [20]. Evidence suggests that stress is associated with unhealthy changes in food seeking behavior including increased consumption of foods high in sugar and fat [67, 68], as well as increased alcohol consumption [69, 70]. Of all types of physical poses, gentle poses would likely exert the most profound relaxation response. Perhaps an effective weight loss intervention would include a combination of active physical poses for their exercise benefits, as well as gentle poses for their possible effects on the HPA axis response to stress, particularly as it relates to self-medicating with food and alcohol. While combination approaches have resulted in weight loss in past studies [36–38], none looked specifically at the combination of active and gentle poses. 

Compared to other components of yoga practice, frequency of philosophy study most often predicted aspects of health. It is doubtful that reading yoga philosophy texts will lead to lower BMI or more happiness. Rather, because individuals who were intense practitioners (≥5 days per week of yoga practice) studied philosophy significantly more often than those who practiced less than once per week, it is likely that frequency of philosophy study served as a “proxy variable” for intense practice. In addition, these same intense practitioners tended to practice all aspects of yoga, with more days of practice per month of all of the physical poses, breath work, and meditation. Thus, any relationship between philosophy study and health may reflect the relationship of frequency and intensity of yoga practice to health. This provides more evidence that an intense practice involving all aspects of yoga practice may be more beneficial to health than a less intense practice that includes only one or two aspects of yoga practice, such as just practicing the physical poses or breath work.

A number of limitations existed in this study. The findings of this study are generalizable only to Iyengar yoga practitioners in the USA. Second, anonymous online surveys have the potential for denial, deception, and recall and/or response bias. Thus, answers for measures such as height and weight might not be accurate. The response rate of 27% was low, which could potentially result in bias. Because subjects were predominantly white, female, and highly educated, it is not known if a lack of diversity may have limited the ability to control for these demographics in the models. Finally, the cross-sectional nature of the study allows one to draw inferences, but do not allow one to conclude that yoga actually impacts health. It should be noted that the use of the word “predicted” when describing the relationship between yoga practice and health is the appropriate term when interpreting linear regressions, but it does not imply causality. Despite these limitations, this study makes an important contribution to understanding of the practice of yoga and its potential contribution to practitioners' health.

## 5. Conclusion

In conclusion, yoga may be a useful intervention for improving health behaviors or life-style-related health conditions. Frequency of home practice appears to be very important—more important than how long an individual has been practicing or how many classes one takes. This emphasizes a simple fact: it is not enough simply to learn how to do healthy behaviors. Rather, healthy behaviors must be incorporated into one's daily life. While these findings suggest that individuals will only glean benefits from yoga practice that are proportional to the energy they are willing to invest in making it a part of their lives, the findings also suggest that they do not have to practice for years in order to reap the rewards. 

What one practices, be it the different types of physical poses, breath work, or meditation, is important because the different aspects of yoga practice may well have different health benefits. Randomized clinical trials are needed to examine causal relationships between the different aspects of yoga practice and aspects of health. For instance, does an intervention focusing on gentle poses positively affect feeding behaviors? Does an intervention focusing on vigorous poses effect sleep better than an intervention focusing on gentle poses? 

While this study focused exclusively on Iyengar yoga, it is important to note that styles of yoga differ in what components of yoga practice are emphasized. Thus, some styles of yoga may be better suited for certain individuals, depending upon the aspects of health they are seeking to improve, as well as their temperament and physical condition. For this reason, future research should examine the comparative effectiveness of different styles of yoga on a variety of health outcomes.

## Figures and Tables

**Table 1:** Demographic characteristics of study sample (*N* = 1045).

Variables	*M* (SD)	Range
Age (*n* = 1043)	51.7 (11.7)	19–87

	Frequency	Percent

Gender		
Female	880	84.2
Race		
White	932	89.2
Other^a^	113	10.8
Marital status		
Married/lives with partner	730	69.8
Single^b^/widowed/separated/divorced	308	29.5
Other	7	0.7
Employment		
Full time	532	50.9
Part time	277	26.5
Not employed	236	22.6
Education		
High school/GED/trade/vocational school/Other	31	3.0
Some college	100	9.6
College graduate	386	36.9
Master's degree	387	37.0
Doctoral degree	141	13.5

^a^Multiracial (*n* = 38; 3.6%), Asian (*n* = 28; 2.7%), African American (*n* = 18; 1.7%), American Indian/Alaskan/Hawaiian/Pacific Islander (6; 0.6%), other (*n* = 23; 2.2%).

^b^Never married.

**Table 2:** Summary of results of final linear and logistic regression models predicting health outcomes from general patterns of yoga practice in combination with influential demographic predictors (*N* = 1045).

Health outcome	Parameter statistics			
	Final predictors^a^	*B*	SE	*t*
Mindfulness	Practice frequency^b^	.106	.014	7.53^∗∗^
	Years of practice	.039	.018	2.17^∗^
Subjective well-being	Practice frequency^b^	.183	.034	5.31^∗∗^
	Gender^c^	3.39	.915	3.72^∗∗^
BMI (*n* = 1034)	Practice frequency^b^	−.043	.012	−3.26^∗∗^
	Gender^c^	−2.013	.321	−6.28^∗∗^
Fruit and vegetables/Day (*n* = 1043)	Practice frequency^b^	.031	.006	5.59^∗∗^
	Age	.013	.005	2.92^∗∗^
	Gender^c^	−.583	.147	−3.97^∗∗^
Sleep disturbance	Practice frequency^b^	−.052	.009	−5.58^∗∗^
Fatigue	Practice frequency^b^	−.171	.042	−4.02^∗∗^
	Age	−.072	.011	−6.36^∗∗^
	Practice frequency^b^ x Age	.002	.001	2.91^∗∗^

				Wald/OR

Vegetarian status	Practice frequency^b^	.056	.011	25.78^∗^/1.057^∗^

^a^Each final model includes all predictors included in the final model. Demographic covariates were included if they had at least a small (*r* = .1) and significant (*P* < .05) correlation with the health variable. No demographic covariate met these criteria that was not included in the final model. ^b^Days per month of home yoga practice. ^c^Gender coded males “0,” females “1.” Abbreviations—*B*: unstandardized beta weight. SE: standard error. *t*: *t* score for linear regressions. x: interaction effect. Wald: Wald statistic for logistic regressions. OR: odds ratio. BMI: body mass index. Note: for all measures, higher scores indicate more of the concept measured. ^∗^ *P* < 0.05 level (2-tailed). ^∗∗^ *P* < 0.01 (2-tailed).

**Table 3:** Results of final linear and logistic regression models predicting health outcomes from specific types of yoga practice^a^ in combination with influential demographic predictors (*N* = 1045).

Health outcome	Final predictors^b^	*B*	S.E.	*t*
Mindfulness	Breath work^c^	1.60	.29	5.51^∗^
	Meditation^c^	1.05	.28	3.71^∗^
	Philosophy study	.31	.08	4.09^∗^
Subjective well-being	Meditation^c^	2.80	.71	3.95^∗^
	Philosophy study	.76	.20	3.87^∗^
	Gender^d^	3.40	.91	3.73^∗^
BMI (*n* = 1034)	Vigorous poses^e^	−.05	.02	−3.27^∗^
	Philosophy study	−.16	.07	−2.22^∗∗^
	Gender^d^	−2.03	.32	−6.34^∗^
Fruit and vegetables/day (*n* = 1043)	Standing poses^e^	.024	.01	3.31^∗^
	Gentle poses^f^	.36	.11	3.16^∗^
	Gender^d^	−.59	.15	−4.03^∗^
	Age	.01	.01	3.17^∗^
Sleep disturbance	Vigorous poses^e^	−.07	.14	−5.50^∗^
Fatigue	Inversions^e^	−.05	.01	−4.62^∗^
	Meditation^c^	−.52	.18	−2.83^∗^
	Age	−.05	.01	−−6.53^∗^

				Wald/OR

Vegetarian status^g^	Gentle poses^f^	.73	.24	9.05^∗^/2.07^∗^
	Philosophy study	.24	.06	15.84^∗^/1.27^∗^
Alcohol consumption^h^	Gentle poses^f^	−.48	.13	14.37^∗^/.621^∗^
	Race^i^	.92	.22	17.56^∗^/.53^∗^

^a^Specific yoga practices: physical poses (standing, vigorous, inversions, and gentle), breath work, meditation, and yoga philosophy study. ^b^Each final model includes all predictors included in the final model. Demographic covariates were included if they had at least a small (*r* = .1) and significant (*P* < .05) correlation with the health variable. No demographic variable met these criteria that was not included in the final model. ^c^≤ or > once per week. ^d^Gender coded “0” = male “1” = female. ^e^Days per month. ^f^ *≤ *or *≥ *30 minutes per week. ^g^Vegetarian status coded no = “0”, yes = “1”. ^h^Alcohol consumption (number of drinks one consumes on a typical day when one drinks) coded ≤2 drinks per day = “0”, *>*2 drinks per day. ^i^Race coded “0” = other, “1” = white. Abbreviations—*B*: unstandardized beta weight. SE: standard error. *t*: *t* score for linear regressions. Wald: Wald statistic for logistic regressions. OR: odds ratio. BMI: body mass index. Note: For all measures, higher scores indicate more of the concept measured. ^∗^ *P* < 0.05 level (2-tailed). ^∗∗^ *P* < 0.01 (2-tailed).
